# MAGE: metafounders-assisted genomic estimation of breeding value, a novel additive-dominance single-step model in crossbreeding systems

**DOI:** 10.1093/bioinformatics/btae044

**Published:** 2024-01-24

**Authors:** Yue Zhuo, Heng Du, ChenGuang Diao, WeiNing Li, Lei Zhou, Li Jiang, JiCai Jiang, JianFeng Liu

**Affiliations:** State Key Laboratory of Animal Biotech Breeding, College of Animal Science and Technology, China Agricultural University, Beijing 100193, China; State Key Laboratory of Animal Biotech Breeding, College of Animal Science and Technology, China Agricultural University, Beijing 100193, China; State Key Laboratory of Animal Biotech Breeding, College of Animal Science and Technology, China Agricultural University, Beijing 100193, China; State Key Laboratory of Animal Biotech Breeding, College of Animal Science and Technology, China Agricultural University, Beijing 100193, China; State Key Laboratory of Animal Biotech Breeding, College of Animal Science and Technology, China Agricultural University, Beijing 100193, China; State Key Laboratory of Animal Biotech Breeding, College of Animal Science and Technology, China Agricultural University, Beijing 100193, China; Department of Animal Science, North Carolina State University, Raleigh, NC 27695, United States; State Key Laboratory of Animal Biotech Breeding, College of Animal Science and Technology, China Agricultural University, Beijing 100193, China

## Abstract

**Motivation:**

Utilizing both purebred and crossbred data in animal genetics is widely recognized as an optimal strategy for enhancing the predictive accuracy of breeding values. Practically, the different genetic background among several purebred populations and their crossbred offspring populations limits the application of traditional prediction methods. Several studies endeavor to predict the crossbred performance via the partial relationship, which divides the data into distinct sub-populations based on the common genetic background, such as one single purebred population and its corresponding crossbred descendant. However, this strategy makes prediction inaccurate due to ignoring half of the parental information of crossbreed animals. Furthermore, dominance effects, although playing a significant role in crossbreeding systems, cannot be modeled under such a prediction model.

**Results:**

To overcome this weakness, we developed a novel multi-breed single-step model using metafounders to assess ancestral relationships across diverse breeds under a unified framework. We proposed to use multi-breed dominance combined relationship matrices to model additive and dominance effects simultaneously. Our method provides a straightforward way to evaluate the heterosis of crossbreeds and the breeding values of purebred parents efficiently and accurately. We performed simulation and real data analyses to verify the potential of our proposed method. Our proposed model improved prediction accuracy under all scenarios considered compared to commonly used methods.

**Availability and implementation:**

The software for implementing our method is available at https://github.com/CAU-TeamLiuJF/MAGE.

## Introduction

In animal breeding and production, it has been a common way to perform crosses between different types of purebreds for fully exploiting heterosis of crossbred population (Dickerson 1973). Although improving crossbred performance is the primary objective in animal production, animal breeders mainly focus on determining the best from purebred candidates based on genetic evaluation (Hartmann 1992, Wientjes and Calus 2017).

Traditional strategies, such as BLUP-based methods, can significantly achieve genetic progress, especially genomic prediction in purebreds. However, purebred genetic improvements might not yield proportionally crossbred improvements (Esfandyari *et al.* 2015). This mainly lies in disparities in allele frequencies, genotype-by-environment interactions, or the measurement of the trait between purebred and crossbred animals (Lutaaya *et al.* 2001). Thus, the genetic correlation between purebred and crossbred performance ($r_{pc}$) being often much smaller than 1 (Wientjes and Calus 2017), resulting in the advantage of genetic evaluation combined purebred and crossbred performance (Wei and van der Werf 1994, Van Grevenhof and Van der Werf 2015).

With the emergence of a large amount of crossbred performance information, it has become a consensus that incorporating crossbred information into genetic evaluation can significantly increase the prediction accuracy of purebreds for crossbred performance. (Wei and van der Werf 1994, Bijma *et al.* 2001). Furthermore, it is straightforward to validate the combination ability among different breeds via modeling non-additive effects, e.g. dominance, for crossbred populations (Charlesworth and Willis 2009). Nonetheless, due to the differing genetic backgrounds and the disparity in variance components between purebred and crossbred populations, genomic prediction methods specific to purebreds cannot be applied to crossbred populations directly (Wei and van der Werf 1994), rendering the integration of purebred and crossbred data a challenging topic.

To address this technical bottleneck, a suit of methods developed prediction models including crossbred and purebred animals by treating purebred and crossbred traits as distinct yet correlated. This strategy was also known as Combined Crossbred and Purebred Selection (CCPS) (Wei and van der Werf 1994). Based on CCPS, some studies use the partial relationship (Misztal *et al.* 2013, Christensen *et al.* 2014) to divide crossbred additive effects into specific breeds with independent origin relating to their purebred parents (García-Cortés and Toro 2006). This method has improved the prediction accuracy of purebred animals (Xiang *et al.* 2016b, Pocrnic *et al.* 2019, Misztal *et al.* 2022). However, it has been pointed out that the partial relationships ignored relationships among diverse breeds, decreasing prediction accuracy to some extent (Xiang *et al.* 2017).

To overcome this limitation of information loss in constructing the partial relationship matrix, we proposed a metafounders-assisted genomic estimation (MAGE) to precisely estimate genetic relationships across distinct breeds. The metafounders method (VanRaden 1992) was employed to evaluate across-population relationships termed ancestral relationships among the foundational population by utilizing marker genotypes (Legarra *et al.* 2015). Multiple studies have indicated the effectiveness of metafounders in the multi-population genomic evaluation (Xiang *et al.* 2017, van Grevenhof *et al.* 2019, Macedo *et al.* 2020). This method offers a new paradigm for utilizing crossbred data (Poulsen *et al.* 2022).

Meanwhile, for the crossbreeding system, integration of dominance effects can potentially enhance the predictive ability of crossbred performance (Varona *et al.* 2022). Although several studies (Ertl *et al.* 2018) have extended traditional single-step genomic prediction methods to include dominance effects in purebred populations, dominance single-step methods for crossbred data still need to be developed. Accordingly, in the current study, we developed a unified single-step genomic prediction model involving dominance and addictive effects under metafounders-based combined partial relationship matrices construction for additive and dominance effects, respectively.

To further validate the performance of the proposed methods herein, we conducted both simulation and real data analyses for genomic evaluation considering crossbreeding systems. Compared with the commonly used methods, our method outperformed the counterparts in prediction accuracy under all scenarios considered as expected. Our study demonstrated that the MAGE methods can evaluate the potential genetic relationship between different breeds or populations, facilitating the assessment of additive effects and heterosis of crossbreeds.

## Methods

Under the framework of partial relationships, the additive and dominance covariance matrices are constructed as combined partial relationship matrices with metafounders.

For the sake of simplicity, our method is presented using a two-way crossbreeding system. We developed multivariate models, each with subscript A, B, or AB referring to purebred population A, B, or crossbred descendants AB, respectively.

(1) $$\begin{array}{cc} y_{A} & =X_{A}b_{A}+Z_{A}a_{A}+W_{A}d_{A}+e_{A}, \end{array}$$

(2) $$y_{B}=X_{B}b_{B}+Z_{B}a_{B}+W_{B}d_{B}+e_{B},$$

(3) $$\begin{array}{cc} y_{\text{AB}} & =X_{\text{AB}}b_{\text{AB}}+Z_{\text{AB}}u_{\text{AB}}+W_{\text{AB}}v_{\text{AB}}+e_{\text{AB}}, \end{array}$$

where the vectors $y_{A}$, $y_{B}$, and $y_{\text{AB}}$ contain the phenotypes observations in models 1–3, the vectors $b_{A}$, $b_{B}$, and $b_{\text{AB}}$ denote the fixed effects for different breeds, and the terms $e_{A}∼N(0,\sigma_{A}^{2}I)$, $e_{B}∼N(0,\sigma_{B}^{2}I)$, and $e_{\text{AB}}∼N(0,\sigma_{\text{AB}}^{2}I)$ are the residual error vectors. The vectors $a_{A}$ and $a_{B}$ are denoted as the purebred additive effects for the corresponding population. The vector $u_{\text{AB}}$ is the crossbred additive effect, which reflects genetic performance for animals when mating across breeds. The vectors $d_{A}$, $d_{B}$, and $v_{\text{AB}}$ are defined similarly for the dominance effects. The matrices $X_{A}$, $X_{B}$, $X_{\text{AB}}$, $Z_{A}$, $Z_{B}$, $Z_{\text{AB}}$, $W_{A}$, $W_{B}$, and $W_{\text{AB}}$ are the incidence matrices in models 1–3.

Considering the disparity in variance components between different breeds, we construct the variance-covariance matrices of additive or dominance effect for each specific breed separately. Thus, the additive and dominance effects are divided into different parts corresponding to each specific breed, such as:

(4) $$\begin{array}{c} u_{\text{AB}}=u_{\text{AB}}^{A}+u_{\text{AB}}^{B}, \end{array}$$

where $u_{\text{AB}}^{A}$ and $u_{\text{AB}}^{B}$ mean the crossbred additive effects inherited from different breeds and can be expressed as $u_{\text{AB}}^{A}=\frac{1}{2}u_{A}+\Phi^{A}$ and $u_{\text{AB}}^{B}=\frac{1}{2}u_{B}+\Phi^{B}$. The effects $u_{A}$ and $u_{B}$ contain parental crossbred additive effects for different breeds, and the vectors $\Phi^{A}$ and $\Phi^{B}$ are the Mendelian sampling effects.

The dominance effects typically result from the interaction between alleles from both purebred populations A and B. Consequently, unlike additive effects, the crossbred dominance effects cannot be decomposed into sub-effects related to the dominance effects of populations A or B. However, these effects can be divided into different components, influenced by the alleles from either populations A or B. We split the crossbred dominance effects $v_{\text{AB}}$ into two breed-specific partial crossbred dominance effects:

(5) $$\begin{array}{c} v_{\text{AB}}=v_{\text{AB}}^{A}+v_{\text{AB}}^{B}. \end{array}$$

where $v_{\text{AB}}^{A}$ and $v_{\text{AB}}^{B}$ denote the breed-specific partial crossbred dominance effects for populations A and B, respectively. Those partial effects are not inherited from the parent populations and are not associated with the dominance effects of the purebred population, which is a crucial distinction from the additive effect formula 4. In addition, these breed-specific partial dominance effects reflect the variability in the combining ability of populations A and B for crossbred dominance effects.

Thus, the model 3 can be written as:

(6) $$\begin{array}{c} y_{\text{AB}}=X_{\text{AB}}b_{\text{AB}}+Z_{\text{AB}}^{A}u_{\text{AB}}^{A}+Z_{\text{AB}}^{B}u_{\text{AB}}^{B}+W_{\text{AB}}^{A}v_{\text{AB}}^{A}+W_{\text{AB}}^{B}v_{\text{AB}}^{B}+e_{\text{AB}}. \end{array}$$

Then, the variance-covariance matrix of additive effect can be written as:

(7) $$\begin{array}{c} \text{Var}\left[\begin{array}{c} a_{A}\\ a_{B}\\ u_{\text{AB}} \end{array}\right]=\text{Var}\left[\begin{array}{c} a_{A}\\ a_{B}\\ u_{\text{AB}}^{A}+u_{\text{AB}}^{B} \end{array}\right]=\text{Var}\left[\begin{array}{c} a_{A}\\ 0\\ u_{\text{AB}}^{A} \end{array}\right]+\text{Var}\left[\begin{array}{c} 0\\ a_{B}\\ u_{\text{AB}}^{B} \end{array}\right], \end{array}$$

where assumes $\text{Cov}\left(u_{\text{AB}}^{A},u_{\text{AB}}^{B}\right)=0$. In order to express the genetic variance-covariance matrix using a Kronecker product, we introduce crossbred genetic effects $u_{A}$ and $u_{B}$. Then, the variance-covariance matrix of additive effect can be formulated as:

(8) $$\begin{array}{c} \begin{array}{c} \text{Var}\left[\begin{array}{c} a_{A}\\ a_{\text{AB}}^{A}\\ u_{A}\\ u_{\text{AB}}^{A} \end{array}\right]=\left[\begin{array}{cc} \sigma_{a}^{2}{\;}_{A,A} & \sigma_{a}^{2}{\;}_{A,\text{AB}}\\ \sigma_{a}^{2}{\;}_{\text{AB},A} & \sigma_{a}^{2}{\;}_{\text{AB},\text{AB}} \end{array}\right]⊗H^{A^{\Gamma }},\\ \text{Var}\left[\begin{array}{c} a_{B}\\ a_{\text{AB}}^{B}\\ u_{B}\\ u_{\text{AB}}^{B} \end{array}\right]=\left[\begin{array}{cc} \sigma_{a}^{2}{\;}_{B,B} & \sigma_{a}^{2}{\;}_{B,\text{AB}}\\ \sigma_{a}^{2}{\;}_{\text{AB},B} & \sigma_{a}^{2}{\;}_{\text{AB},\text{AB}} \end{array}\right]⊗H^{B^{\Gamma }}, \end{array} \end{array}$$

where the vectors $a_{\text{AB}}^{A}$ and $a_{\text{AB}}^{B}$ contain two artificial random effects, and the term $\sigma_{a}^{2}$ denotes the additive covariance. The matrices $H^{A^{\Gamma }}$ and $H^{B^{\Gamma }}$ are defined as the combined additive partial relationship matrices using metafounders.

Similarly, the variance-covariance matrix of dominance effect can be expressed as:

(9) $$\begin{array}{c} \begin{array}{c} \text{Var}\left[\begin{array}{c} d_{A}\\ d_{\text{AB}}^{A}\\ v_{A}\\ v_{\text{AB}}^{A} \end{array}\right]=\left[\begin{array}{cc} \sigma_{d}^{2}{\;}_{A,A} & \sigma_{d}^{2}{\;}_{A,\text{AB}}\\ \sigma_{d}^{2}{\;}_{\text{AB},A} & \sigma_{d}^{2}{\;}_{\text{AB},\text{AB}} \end{array}\right]⊗T^{A},\\ \text{Var}\left[\begin{array}{c} d_{B}\\ d_{\text{AB}}^{B}\\ v_{B}\\ v_{\text{AB}}^{B} \end{array}\right]=\left[\begin{array}{cc} \sigma_{d}^{2}{\;}_{B,B} & \sigma_{d}^{2}{\;}_{B,\text{AB}}\\ \sigma_{d}^{2}{\;}_{\text{AB},B} & \sigma_{d}^{2}{\;}_{\text{AB},\text{AB}} \end{array}\right]⊗T^{B}, \end{array} \end{array}$$

where the terms $d_{\text{AB}}^{A}$, $d_{\text{AB}}^{B}$, and $\sigma_{d}^{2}$ are similar to $a_{\text{AB}}^{A}$, $a_{\text{AB}}^{B}$, and $\sigma_{a}^{2}$. Then, the matrices $T^{A}$ and $T^{B}$ are the combined dominance partial relationship matrices using metafounders, and those dominance relationships come only from the ancestral relationship between populations.

In the following sections, we take the matrices $H^{A^{\Gamma }}$ and $T^{A}$ as an example to describe the method of constructing of the variance-covariance matrices of additive or dominance effects, and the matrices $H^{B^{\Gamma }}$ and $T^{B}$ can be constructed similarly.

### Constructing combined additive partial relationship matrix

Similar to traditional single-step methods (Legarra *et al.* 2009), the inverse of $H^{A^{\Gamma }}$ is defined as follows:

(10) $$\begin{array}{c} {\left(H^{A^{\Gamma }}\right)}^{-1}=\left[\begin{array}{cc} 0 & 0\\ 0 & {\left(\left(1-\omega \right)G^{A}+\omega A_{22}^{A^{\Gamma }}\right)}^{-1}-{\left(A_{22}^{A^{\Gamma }}\right)}^{-1} \end{array}\right]+{\left(A^{A^{\Gamma }}\right)}^{-1}, \end{array}$$

where the matrices $A^{A^{\Gamma }}$ and $G^{A}$ represent the pedigree- and marker-based additive partial relationship matrices using metafounders (Legarra *et al.* 2015), respectively. The matrix $A_{22}^{A^{\Gamma }}$, in which all animals are genotyped, is a submatrix of $A^{A^{\Gamma }}$. The term ω denotes the relative weight of polygenic additive effects.

#### Constructing pedigree-based additive partial relationship matrix

The matrix $A^{A^{\Gamma }}$ can be derived from the traditional additive partial relationship matrix $A^{A}$. In two-way crossbreeding systems, the matrix $A^{A^{\Gamma }}$ can be expressed using the following formula, and derivation details are provided in Supplementary Appendix A:

(11) $$\begin{array}{c} A^{A^{\Gamma }}=A^{A}\left(1-\frac{1}{2}\Gamma_{A}\right)+K^{A}\cdot \Gamma_{A}+Q_{\text{AB}}^{A}\cdot \Gamma_{\text{AB}}^{A}, \end{array}$$

where the recursive formulas for the element in $A^{A}$ are:

(12) $$\begin{array}{c} a_{ii}^{A}=f_{i}^{A}+\frac{1}{2}a_{f(i)m(i)}^{A},\\ a_{ij}^{A}=\frac{1}{2}\left(a_{f(i)j}^{A}+a_{m(i)j}^{A}\right), \end{array}$$

where the element $a_{ij}^{A}$ signifies the additive partial relationship between animals *i* and *j*, and animals *j* is not a descendant of *i*. Additionally, the terms f(i) and m(i) represent the parents of animal *i*, and the term $f_{i}^{A}$ denotes the proportion of ancestry from breed A for animal *i*.

Then, terms $\Gamma_{A}$ and $\Gamma_{\text{AB}}^{A}$ denote within- and across-breed ancestral relationships, respectively. The within-breed ancestral relationship $\Gamma_{A}$ is similar to that in the relationships with metafounders for purebred animals. The purebred relationships with metafounders are given by $a_{ij}^{\Gamma }=a_{ij}\left(1-\frac{1}{2}\Gamma_{A}\right)+\Gamma_{A}$, where $a_{ij}$ is the traditional relationships between the animals *i* and *j*, and both animals are from breed A (Legarra *et al.* 2015). The across-breed ancestral relationship $\Gamma_{\text{AB}}^{A}$ is usually defined as the background relationships between animals of two different breeds. For the data incorporating purebred and crossbred animals, we uses the matrices $K^{A}$ and $Q_{\text{AB}}^{A}$ in formula 11 to construct $A^{A^{\Gamma }}$:

(13) $$\begin{array}{c} K^{A}=\left[\begin{array}{cc} 1_{n\times n} & {\frac{1}{2}}_{n\times m}\\ {\frac{1}{2}}_{m\times n} & {\frac{1}{4}}_{m\times m} \end{array}\right],\\ Q_{\text{AB}}^{A}=\left[\begin{array}{cc} 0_{n\times n} & {\frac{1}{2}}_{n\times m}\\ {\frac{1}{2}}_{m\times n} & {\frac{1}{2}}_{m\times m} \end{array}\right], \end{array}$$

where the matrix $1_{n\times n}$ is a n×n matrix of 1’s, and the subscripts *n* and *m* represent the number of purebred and crossbred animals, respectively.

#### Constructing marker-based additive partial relationship matrix

Moreover, the marker-based additive partial relationship matrix $G^{A}$ is constructed as:

(14) $$\begin{array}{c} G^{A}=G_{A}^{A}\;+G_{\text{AB}}^{A}\\ =\frac{Z^{A}Z^{\text{AT}}}{s^{A}}\;+\left[\begin{array}{cc} 0 & Z_{A}^{A}Z_{\text{AB}}^{B}{\;}^{T}\\ Z_{\text{AB}}^{B}Z_{A}^{A}{\;}^{T} & Z_{\text{AB}}^{A}Z_{\text{AB}}^{B}{\;}^{T}+Z_{\text{AB}}^{B}Z_{\text{AB}}^{A}{\;}^{T} \end{array}\right]/\left(2s^{A}\right), \end{array}$$

where the term $s^{A}$ is the scaling parameter and will be described in the following subsection. For different breeds, the elements of the matrix $Z^{A}$ are defined as:

(15) $$\begin{array}{c} z_{ij}^{A}=\{\begin{array}{cc} m_{ij}^{A}-2p_{j}^{A}, & \;\text{for}\;\text{purebred}\;\text{animals}\\ m_{ij}^{A}-p_{j}^{A}, & \;\text{for}\;\text{crossbred}\;\text{animals} \end{array} \end{array}$$

where the element $p_{j}^{A}$ is the specific allele frequencies from breed A for SNP *j*, which is based on marker genotypes for purebred animals and specific marker alleles from breed A for crossbred animals. For purebred animals, the element $m_{ij}^{A}$ is:

(16) $$\begin{array}{c} m_{ij}^{A}=\{\begin{array}{ccc} 2, & \;\text{for}\;\text{genotypes} & A_{1}A_{1}\\ 1, & \;\text{for}\;\text{genotypes} & A_{1}A_{2}\\ 0, & \;\text{for}\;\text{genotypes} & A_{2}A_{2} \end{array} \end{array}$$

and for crossbred animals, the element $m_{ij}^{A}$ is:

(17) $$\begin{array}{c} m_{ij}^{A}=\{\begin{array}{ccc} 1, & \;\text{for}\;\text{genotypes} & A_{1}\\ 0, & \;\text{for}\;\text{genotypes} & A_{2} \end{array} \end{array}$$

where the elements $m_{ij}^{A}$ for crossbred animals are based on specific marker alleles from breed A for crossbred animals. Consequently, the genome phasing is essential to determine the specific marker alleles from breed A.

The matrices $Z_{A}^{A}$ and $Z_{\text{AB}}^{A}$ in formula 14 are similar to $Z^{A}$, while the matrix $Z_{A}^{A}$ only consists of breed A animals and the matrix $Z_{\text{AB}}^{A}$ only consists of crossbred AB animals. The matrix $Z_{\text{AB}}^{B}$ also consists of crossbred AB animals, but based on marker genotypes from breed B.

#### Compatibility adjustment of the addictive relationship matrix

In order to achieve compatibility of pedigree- or marker-based relationship matrices (Legarra *et al.* 2015) and determine parameters $\Gamma_{A}$, $\Gamma_{\text{AB}}^{A}$, and $s^{A}$, we employ a method which enforces equivalence between the expected changes in the mean and variance for these matrices (Searle and Khuri 2017). In two-way crossbreeding systems,

(18) $$\begin{array}{c} s^{A}=(\left(\bar{K_{22}^{A}}-\bar{A_{22}^{A}}/2\right)\left(\bar{\mathrm{diag}G_{A}^{A}}+\bar{\mathrm{diag}G_{\text{AB}}^{A}}-\frac{\bar{\mathrm{diag}Q_{22}^{A}}}{\bar{Q_{22}^{A}}}\bar{G_{\text{AB}}^{A}}\right)\\ -\left(\bar{K_{22}^{A}}-\bar{\mathrm{diag}A_{22}^{A}}/2\right)\bar{G_{A}^{A}})/\left(\bar{\mathrm{diag}A_{22}^{A}K_{22}^{A}}-\bar{\mathrm{diag}K_{22}^{A}A_{22}^{A}}\right),\\ \Gamma_{A}=\frac{\bar{G_{A}^{A}}/s^{A}-\bar{A_{22}^{A}}}{\bar{K_{22}^{A}}-\bar{A_{22}^{A}}/2},\\ \Gamma_{\text{AB}}^{A}=\frac{G_{\text{AB}}^{A}/s^{A}}{Q_{22}^{A}}. \end{array}$$

### Constructing combined dominance partial relationship matrix

Consistent with traditional purebred dominance method (Mota *et al.* 2019), the inverse of $T^{A}$ can be expressed as:

(19) $$\begin{array}{c} {\left(T^{A}\right)}^{-1}=\left[\begin{array}{cc} 0 & 0\\ 0 & {\left(\left(1-\omega \right)M^{A}+\omega D_{22}^{A}\right)}^{-1}-{\left(D_{22}^{A}\right)}^{-1} \end{array}\right]+{\left(D^{A}\right)}^{-1}, \end{array}$$

where the matrices $D^{A}$ and $M^{A}$ represent the pedigree- and marker-based dominance partial relationship matrices, respectively. The matrix $D_{22}^{A}$, in which all animals are genotyped, is a submatrix of $D^{A}$. The term ω signifies the relative weight on polygenic dominance effects (Christensen and Lund 2010).

#### Constructing pedigree-based dominance partial relationship matrix

Dominance relationships are typically assessed as the proportion of shared genotypes between animals (Cockerham 1954).

In two-way crossbreeding systems, extending dominance relationships to the crossbred population is easy. The dominance partial relationship matrix $D^{A}$ can be written as:

(20) $$\begin{array}{c} d_{ii}^{A}=f_{i}^{A}-\frac{1}{2}a_{f(i)m(i)},\\ d_{ij}^{A}=f_{ij}^{A}\frac{a_{f(i)f(j)}a_{m(i)m(j)}+a_{f(i)m(j)}a_{m(i)f(j)}}{4}, \end{array}$$

where the term $f_{i}^{A}$ denotes the proportion of ancestry from breed A for animal *i*, and the term $a_{f(i)m(i)}$ signifies the traditional additive relationship between animals f(i) and m(i). Additionally, the terms f(i) and m(i) represent the parents of animal *i*. The term $f_{ij}^{A}$ represents the mean of the proportion of ancestry from breed A for animals *i* and *j*, and $f_{ij}^{A}=\frac{f_{i}^{A}+f_{j}^{A}}{2}$.

In particular, several methods are available for handling diagonal elements of the dominance relationship matrix of purebred animals. One such method is the natural scenario approach, which calculates the diagonal elements using the identical formula applied to the off-diagonal elements. In addition, the diagonal elements can be set to $d_{ii}=1$ (Cockerham 1954) or $d_{ii}=1-F_{i}$ (Ovaskainen *et al.* 2008), where $F_{i}$ is the inbreeding coefficient and can be calculated as $\frac{1}{2}a_{f(i)m(i)}$. The formula $d_{ii}=1-F_{i}$, known as the gene dropping approach, can be explained as the variance acquired by an animal due to inbreeding in the additive effects (i.e. $1+F_{i}$) will be offset in dominance (i.e. $1-F_{i}$) (Kempthorne 1957, Ovaskainen *et al.* 2008). In light of the gene dropping approach demonstrating superior accuracy and lower deviations relative to other methods (Mota *et al.* 2019), we define the diagonal elements of the dominance partial relationship matrix as $d_{ii}^{A}=f_{i}^{A}-\frac{1}{2}a_{f(i)m(i)}$.

#### Constructing marker-based dominance partial relationship matrix

The marker-based dominance partial relationship matrix $M^{A}$ is defined based on the covariance of the dominant deviations:

(21) $$\begin{array}{c} M^{A}=\frac{W^{A}W^{\text{AT}}}{\sum {\left(2p_{j}^{A}q_{j}^{A}\right)}^{2}}. \end{array}$$

For breed A, the matrix $W^{A}$ contains elements $-2{\left(q_{j}^{A}\right)}^{2}$, $2p_{j}^{A}q_{j}^{A}$, or $-2{\left(p_{j}^{A}\right)}^{2}$, depending on whether the SNP *j* of animal *i* is $A_{1}A_{1}$, $A_{1}A_{2}$, or $A_{2}A_{2}$, respectively. For crossbreeds AB, both specific marker alleles from breed A and the genotype of the crossbred animal must be considered:

(22) $$\begin{array}{c} \begin{array}{c} w_{ij}=\{\begin{array}{ccc} -\left({\left(q_{j}^{A}\right)}^{2}+q_{j}^{A}q_{j}^{B}\right)/2, & \;if & A_{1}\left(A_{1}A_{1}\right)\\ \left(p_{j}^{A}q_{j}^{A}+q_{j}^{A}p_{j}^{B}\right)/2, & \;if & A_{1}\left(A_{1}A_{2}\right)\\ \left(p_{j}^{A}q_{j}^{A}+p_{j}^{A}q_{j}^{B}\right)/2, & \;if & A_{2}\left(A_{2}A_{1}\right)\\ -\left({\left(p_{j}^{A}\right)}^{2}+p_{j}^{A}p_{j}^{B}\right)/2, & \;if & A_{2}\left(A_{2}A_{2}\right) \end{array} \end{array} \end{array}$$

where the term $A_{1}\left(A_{1}A_{1}\right)$ indicates that the genotypes of animal *j* is $A_{1}A_{2}$ and the allele $A_{1}$ is specific marker alleles from breed A. Consequently, the genome phasing is essential to determine the specific marker alleles from breed A for crossbred animals.

Accordingly, we developed a trivariate additive-dominance model incorporating purebred and crossbred using metafounders. The model can be easily extended to the three- or four-way crossbreeding systems commonly employed in pork production. Calculation of the matrices $H^{A^{\Gamma }}$, $H^{B^{\Gamma }}$, $T^{A}$, and $T^{B}$ was achieved by our self-developed program which is available at https://github.com/CAU-TeamLiuJF/MAGE. The genomic prediction of breeding values and dominance effects for purebred and crossbred performance was performed straightforwardly using our previously developed program PIBLUP (Kang *et al.* 2018).

## Results

The effectiveness of our method was validated using both simulated and real data. In our study, the combined partial relationship matrices were computed with MAGE and breeding values were calculated using our previously developed tool PIBLUP (Kang *et al.* 2018). We compared our method to the Christensen proposed one for crossbred data analyses (Poulsen *et al.* 2022). Additionally, the traditional single-step method was employed as a counterpart.

### Simulation study for predicting purebred performance

Population and genomic architecture were simulated using a two-way crossbreeding system using QMSim (Sargolzaei and Schenkel 2009). Traits were simulated at a constant narrow-sense heritability of 0.1. The proportion of dominance, defined as the ratio of dominance variance to genotypic variance, was simulated at 0%, 10%, 30%, or 50%. Ten replicates were performed for the simulation. Further details of the simulation can be found in Supplementary Appendix B.

Following the simulation, 9000 purebred animals and 9000 crossbred animals from three generations were selected as the reference population. Moreover, 50% of those animals were genotyped with 49 111 SNPs. Then, 30% of genotyped animals in the last purebred generation were selected as the validation population.

We employed CCPS and MAGE (i.e. our method) to predict breeding values of purebred animals. The traditional single-step method was employed as a counterpart using either purebred data (ssPB) or purebred-crossbred data (ssCB).

The prediction accuracy was defined as the correlation ($r_{\mathrm{TBV},\mathrm{EBV}}$) between true breeding values (TBV) and estimated breeding values (EBV). Unbiasedness was defined as $\left|1-b_{\mathrm{TBV},\mathrm{EBV}}\right|$, where $b_{\mathrm{TBV},\mathrm{EBV}}$ represents the regression of TBV on EBV. Dominance effects were not considered for evaluating purebred genomic prediction, as only additive effects can be transmitted to offspring.

**Figure 1.**
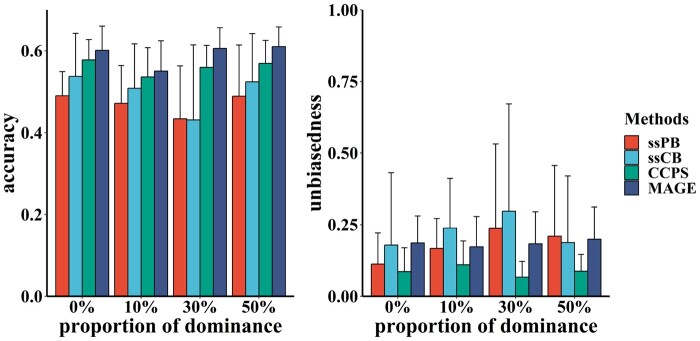
The prediction accuracy and unbiasedness using simulated data for predicting purebred performance using the Christensen method (CCPS), our method (MAGE), the traditional single-step method using purebred data (ssPB), and the traditional single-step method using purebred-crossbred data (ssCB). The proportion of dominance from 0 to 50% was analyzed.

Figure 1 displays the means and standard deviations of prediction accuracy and unbiasedness in the simulation study for predicting purebred performance. The results summarize ten replicates for all proportions of dominance. Accuracies ranged from 0.43 to 0.49 with ssPB, 0.43 to 0.53 with ssCB, 0.53 to 0.57 with CCPS, and 0.55 to 0.61 with MAGE for all cases.

Our method, MAGE, demonstrated the best performance, improving accuracy by 3.2% compared to CCPS. Both CCPS methods, CCPS and MAGE, significantly outperformed the traditional single-step method. The traditional ssCB also improved accuracy, albeit with more significant variance.

However, for all cases, unbiasedness with MAGE, ranging from 0.07 to 0.11, was inferior to CCPS, which ranged from 0.17 to 0.19. Unbiasedness with the traditional single-step method exhibited a wider variation, ranging from 0.11 to 0.24 with ssPB and 0.18 to 0.30 with ssCB.

### Simulation study for predicting crossbred performance

In this simulation study, the simulated data and methods were identical to the simulation study for predicting purebred performance. However, animals from the last crossbred generation were employed as the validation population instead of the purebred generation.

Consequently, the method ssPB was excluded as it did not involve crossbred data. True genotypic values were used for crossbred evaluation, encompassing additive and dominance effects.

**Figure 2.**
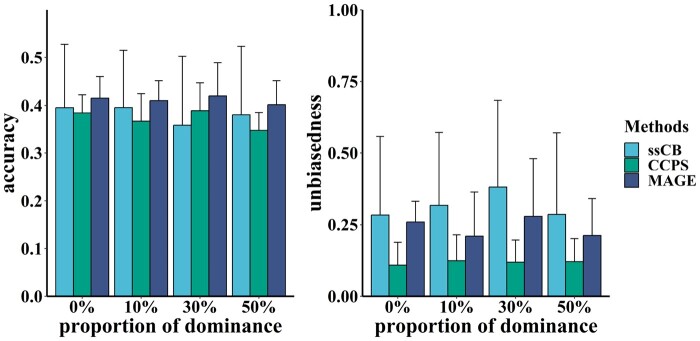
The prediction accuracy and unbiasedness using simulated data for predicting crossbred performance using the Christensen method (CCPS), our method (MAGE), and the traditional single-step method using purebred-crossbred data (ssCB). The proportion of dominance from 0 to 50% was analyzed.

Figure 2 displays the mean and standard deviations of prediction accuracy and unbiasedness in the simulation study for predicting crossbred performance, summarizing the results of 10 replicates across all proportions of dominance. For all cases, accuracies ranged from 0.34 to 0.39 with CCPS and 0.40 to 0.42 with MAGE. The counterpart, which employed the traditional single-step method, exhibited a range of 0.01 to 0.59 and a mean of 0.382.

Our MAGE method achieved a 3.9% improvement in accuracy compared to CCPS. Although the mean accuracy with ssCB surpassed that of CCPS, more significant variation was observed with ssCB. Moreover, the accuracy in the simulation study for predicting crossbred performance was lower than that for predicting purebred performance for all methods and cases.

Similarly, unbiasedness with MAGE was inferior to CCPS’s, while ssCB exhibited the worst performance. Unbiasedness ranged from 0.01 to 0.99, 0.11 to 0.12, and 0.20 to 0.28 with ssCB, CCPS, and MAGE, respectively.

### Real data analyses for predicting purebred performance

In the real data analyses, Beijing Zhongyu Breeding Pig Co., Ltd provided real pig reproduction data. The populations consisted of Yorkshire pigs (YY) and their crossbred offspring (YL) with Landrace pigs (LL). 1,324 total number born (TNB) records from 698 YY and 362 TNB records from 168 YL were used. Among these animals, 333 YY and 131 YL were genotyped with the Illumina PorcineSNP60 Genotyping BeadChip. After quality control, 50 904 SNPs were available in purebred and crossbred populations.

An adjusted repeatability model was employed to predict breeding values, incorporating parity and herd-year-season effects. The relationship matrix was replaced according to the corresponding method. The breed-specific marker alleles in crossbred populations were phased using AlphaPhase (Hickey *et al.* 2011). The metafounders parameter was estimated with MAGE and the result was $s^{A}=1.0479$ and $\Gamma_{A}=0.3944$. The traditional single-step method served as the counterpart, utilizing either purebred data (ssPB) or purebred-crossbred data (ssCB). For the validation group, 30% of purebred animals with phenotypes in the last generation were randomly sampled. As TBV were unavailable, adjusted phenotype values (APV) were used. Ten random validations were performed.

**Figure 3.**
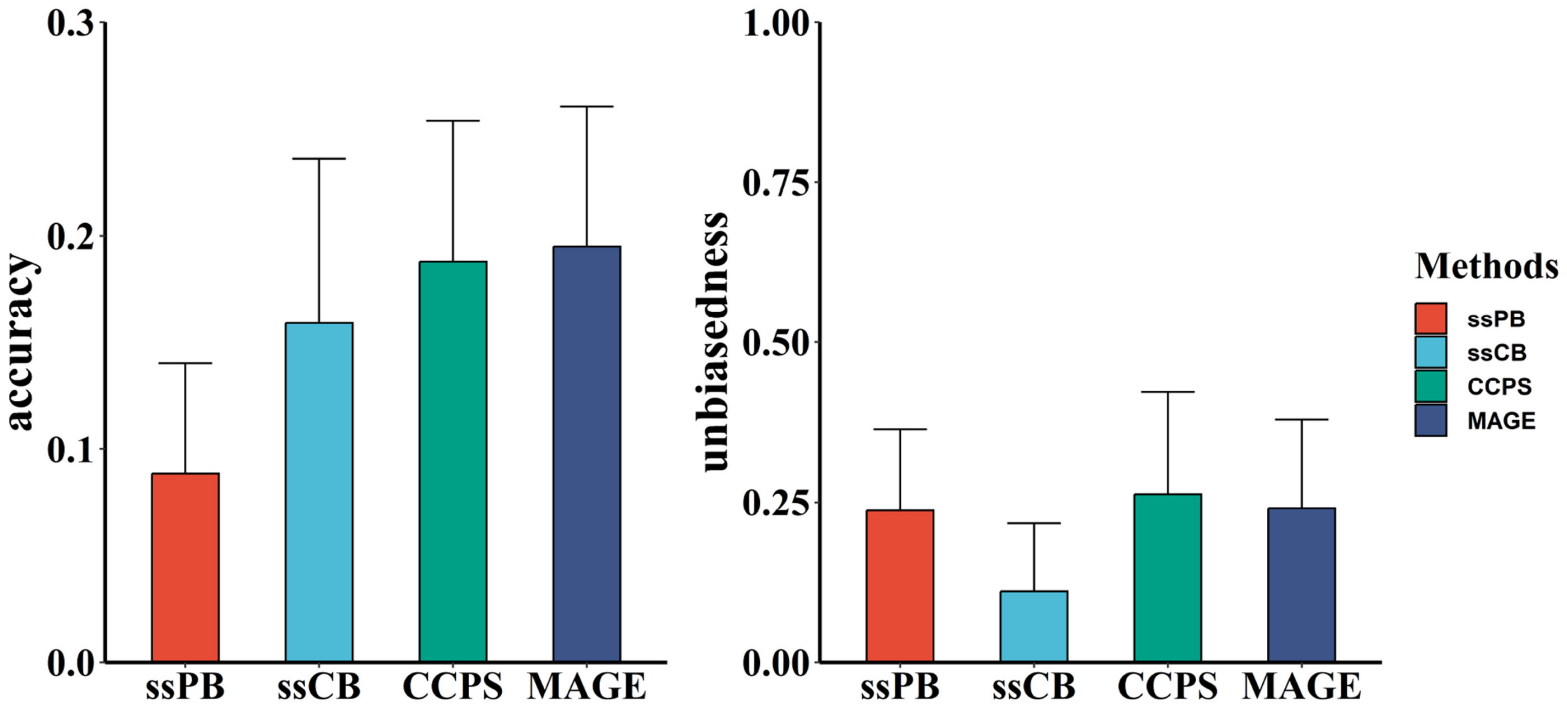
The prediction accuracy and unbiasedness using real data for predicting purebred performance using the Christensen method (CCPS), our method (MAGE), the traditional single-step method using purebred data (ssPB), and the traditional single-step method using purebred-crossbred data (ssCB).

Figure 3 displays the means and standard deviations of prediction accuracy and unbiasedness for predicting purebred performance, summarizing the results of ten random validations. Overall, accuracies ranged from 0.01 to 0.17 with ssPB, 0.03 to 0.31 with ssCB, 0.11 to 0.34 with CCPS, and 0.12 to 0.35 with MAGE. The accuracy of MAGE gained an average of 1.0% improvement compared to CCPS.

Contrary to the simulation, unbiasedness with MAGE was better than that of CCPS, with MAGE achieving an average improvement of 2.3%. However, it is essential to note that genetic prediction using real data, in terms of accuracy and unbiasedness, exhibits more significant standard errors than simulated data for predicting purebred performance.

## Discussion

This study aims to develop a highly efficient method for purebred genetic evaluation utilizing purebred and crossbred data to increase the predictive ability of the breeding value. We propose an extension of partial relationships integrating metafounders to assess ancestral relationships across diverse breeds. Then, dominance relationships were constructed to predict the heterosis for purebred and crossbred populations. Although our method is presented using a two-way crossbreeding system, it is still effective for three-way or more complex crossbreeding systems, and the formulas for complex crossbreeding systems are provided in Supplementary Appendix C. Results from simulated and real data indicate that our new method yields more accurate genomic predictions for purebred and crossbred performance.

As anticipated, both simulated and real validation results demonstrate our method’s superiority in prediction accuracy. We perform one crossbred method (CCPS) and two traditional methods (ssPB, ssCB) to validate the effectiveness of our method. We merely compared our MAGE and CCPS for crossbred methods since CCPS has been demonstrated as the best in previous reports among existing methods in relevant studies (Poulsen *et al.* 2022).

Compared to CCPS, our method improves the prediction accuracy of purebred performance by 3.2% and 1.0% using simulated (Fig. 1) and real data (Fig. 3), respectively. Our method achieves a 3.9% improvement for crossbred performance using simulated data (Fig. 2). Results obtained from real data are less favorable than those from simulated data, possibly due to the lower proportions of dominance in the data (Esfandyari *et al.* 2015) or the smaller number of crossbred animals (Wei and van der Werf 1994).

These findings suggest that our method can be a superior tool for integrating crossbred data into genomic prediction. Our method expands upon CCPS by integrating partial relationships and metafounders. Although partial relationships are valid for crossbred data (Poulsen *et al.* 2022), it is essential to recognize that partial relationships assume no across-breed relationships exist, decreasing prediction accuracy. Consequently, we reconstructed the partial relationships, considering the within- and across-breed ancestral relationships using marker genotypes. Although our method is more challenging than the Christensen method, it incorporates additional information into the models that may enhance genomic prediction capabilities.

Additionally, we examined the effects of incorporating crossbred animals into the reference population (Figs 1–3). The results of purebred methods (ssPB, ssCB) indicate that the prediction accuracy can be improved using crossbred data, even if purebred methods are unsuitable for crossbred data. Given the larger reference population, these findings are understandable, which aligns with conclusions from previous studies (VanRaden 1992, Lutaaya *et al.* 2001). However, some studies have also demonstrated that the accuracy of purebred methods using crossbred data may be lower (Wei and van der Werf 1994). Our study reveals considerable variation with the traditional purebred method using crossbred data (Fig. 1). This result suggests that the traditional purebred method may not reliably predict crossbred performance, even if it occasionally achieves higher accuracy than the crossbred method (Fig. 2). In summary, a suitable crossbred model, such as our method, is better for genetic evaluation of purebred and crossbred performance.

It should be noted that the prediction accuracy of crossbred performance using either method is significantly lower than that of purebred performance (Figs 1 and 2). This result can be attributed to the more complex genetic background of the crossbred population since the crossbred genetic effect is inherited from both parents (García-Cortés and Toro 2006). Furthermore, compared to CCPS, our method demonstrates higher relative advantages in crossbred prediction than in purebred prediction. A possible explanation is that dominance variance, which is more prominent in crossbred populations (Aliloo *et al.* 2016, Class and Brommer 2020), is estimated by our method but ignored by CCPS (Christensen *et al.* 2014). Our method provides the estimated genotypic values (EGV) of both purebred and crossbred animals. The EGV, including additive and dominance effects, can be used to predict heterosis more accurately. Considering the difficulty in estimating epistatic effects, only additive and dominance effects were included in the EGV provided by our model. Thus, the heterosis can be measured by the difference between the individual crossbred EGV and the mean purebred EGV of the parents. The EGV can predict heterosis more accurately since the dominance effects play an important role in the heterosis. The lowest prediction accuracy using real data may be due to the small population size, contributing to a more significant standard error (Fig. 3). As more crossbred phenotypes and genotypes are accumulated, further studies using real data will be necessary to validate the advantages of our method.

Predictions using simulated data indicate that our method results in worse unbiasedness, whether for purebred or crossbred prediction (Figs 1 and 2). This result may be due to the influence of evaluating dominance effects (Zeng *et al.* 2013). Another possibility is that the additive-dominance covariance is overlooked by our method (Fernandez *et al.* 2017). However, our method using real data exhibits better unbiasedness, although both methods have more significant standard errors (Fig. 3). This result suggests that the datasets influence different unbiasedness levels. Nevertheless, the unbiasedness of the traditional single-step method can perform better and is highly variable, implying that it may not be suitable for handling purebred-crossbred data. Overall, this is not a serious concern, as the unbiasedness of both crossbred methods in all scenarios is similar and remains within an acceptable range (Vitezica *et al.* 2013). Still, it is necessary to study the specifics of unbiasedness in more datasets.

The proportions of dominance ranging from 0% to 50% are simulated, as various studies have reported that the proportion of dominance for different traits ranges from 5% to 40% (Norris *et al.* 2006, Serenius *et al.* 2006, Aliloo *et al.* 2016, Xiang *et al.* 2016a, Class and Brommer 2020). Our method exhibits improved accuracy compared to CCPS (Figs 1 and 2), which can be attributed to the evaluation of dominance effects, as noted in some studies (Zeng *et al.* 2013, Sun *et al.* 2014, Esfandyari *et al.* 2016). The accuracy at a large proportion of dominance (e.g. 30% or 50%) is higher than that at a lower proportion (e.g. 0%). However, an approximate linear association between the accuracy and the proportion of dominance, as reported in previous research (Esfandyari *et al.* 2015), may not be evident in this study. Simultaneously, several studies have revealed that dominance effects can not increase predictive ability (Su *et al.* 2012, Vitezica *et al.* 2013). Assessing the role of dominance effects in the crossbred genetic prediction remains an important topic for future research.

Additionally, several adjustments can be applied to our method. For instance, specific algorithms can compute breed proportions using markers instead of pedigree (Calus *et al.* 2022), which may be appropriate for complex crossbreeding systems. Also, pedigree- and marker-based dominance relationships can be measured using various approaches (Cockerham 1954, Ovaskainen *et al.* 2008, Su *et al.* 2012), which are equivalent and differ in variance interpretation. Prior research suggests that the approach used by our method is similar in explaining phenotypes compared to other approaches, while it provides directly estimated breeding values (Vitezica *et al.* 2013).

In conclusion, our method offers a strategy for predicting genetic performance using application prospects in crossbreeding populations, such as pigs, poultry, mice, wheat, and corn. Although this study uses a two-way crossbreeding system for description and validation, our approach can accommodate more complex cases. Individuals mating within crossbred populations, which may be more common in beef production, can also be analyzed by our method. Our method introduces a new relationship for multi-breed populations. It can be applied wherever relationships across breeds may be utilized, such as genome-wide association studies (GWAS) involving multiple breeds. Furthermore, our method can be helpful for populations with different genetic variances. The genetic performance, predicted by mixed linear methods using our relationship, may provide a new approach to correct population stratification of complex crossbreeding populations for GWAS.

## Supplementary Material

btae044_Supplementary_Data

## Data Availability

The data underlying this article will be shared on reasonable request to the corresponding author (liujf@cau.edu.cn).
