# Supplementary material for: MAGE: metafounders-assisted genomic estimation of breeding value, a novel additive-dominance single-step model in crossbreeding systems
Source: Bioinformatics. 2024 Jan 24;40(2):btae044. doi: 10.1093/bioinformatics/btae044 (PMC11212483; doi:10.1093/bioinformatics/btae044)
Supplement: btae044_Supplementary_Data [file btae044_supplementary_data.pdf]

Supplementary Material

Appendix A. The derivation of the pedigree-based additive partial relationship matrix

This appendix presents the derivation of the pedigree-based additive partial relationship matrix with metafounders.

A. Legarra et al. demonstrated that the relationships with metafounders for purebred animals are given by $a_{ij}^{\Gamma} = a_{ij}\left(1 - \frac{1}{2}\Gamma_{\mathrm{A}}\right) + \Gamma_{\mathrm{A}}$ (Legarra et al. 2015), where both animals i and j are from breed A. When animal i is from breed A and animal j is from breed B, $a_{ij}^{\Gamma} = \Gamma_{\mathrm{AB}}$. The aforementioned a_{ij} is the traditional relationships, while Γ_{A} and denote Γ_{AB} within- and across-breed ancestral relationships, respectively.

Consequently, the pedigree-based additive partial relationship matrix $\mathbf{A}^{\mathrm{A}}$ can be expressed as a 2×2 block matrix based on the breeds of animals:

$$\mathbf{A}^{\mathrm{A}} = \begin{bmatrix} \mathbf{A}_{\mathrm{A,A}}^{\mathrm{A}} & \mathbf{A}_{\mathrm{A,AB}}^{\mathrm{A}} \\ \mathbf{A}_{\mathrm{AB,A}}^{\mathrm{A}} & \mathbf{A}_{\mathrm{AB,AB}}^{\mathrm{A}} \end{bmatrix}, \tag{1}$$

where the subscripts of the submatrix indicate the breeds within the covariance matrix. The derivation can be presented separately for different blocks.

The formula for $\mathbf{A}_{\mathrm{A,A}}^{\mathrm{A}^{\Gamma}}$ is straightforward since $\mathbf{A}_{\mathrm{A,A}}^{\mathrm{A}}$ consists of purebred:

$$a_{\mathrm{A,A}_{ij}}^{\mathrm{A}^{\Gamma}} = a_{\mathrm{A,A}_{ij}}^{\mathrm{A}}\left(1 - \frac{1}{2}\Gamma_{\mathrm{A}}\right) + \Gamma_{\mathrm{A}}. \tag{2}$$

Given that $\mathbf{A}_{\mathrm{A,AB}}^{\mathrm{A}}$ contains only off-diagonal elements, the formula for $\mathbf{A}_{\mathrm{A,AB}_{ij}}^{\mathrm{A}^{\Gamma}}$ can be descried as:

$$a_{\mathrm{A,AB}_{ij}}^{\mathrm{A}^{\Gamma}} = \frac{1}{2}\left(a_{\mathrm{A,AB}_{f(i)j}}^{\mathrm{A}^{\Gamma}} + a_{\mathrm{A,AB}_{m(i)j}}^{\mathrm{A}^{\Gamma}}\right), \tag{3}$$

where this formula assumes that animal i is from crossbred AB, while animal j is from breed A. The animals $f(i)$ and $m(i)$ are from breed A and B, respectively.

Thus, it is evident that

$$a_{\mathrm{A,AB}_{m(i)j}}^{\mathrm{A}^{\Gamma}} = \Gamma_{\mathrm{AB}}^{\mathrm{A}} \tag{4}$$

and

$$a_{\mathrm{A,AB}_{ij}}^{\mathrm{A}^{\Gamma}} = a_{\mathrm{A,AB}_{ij}}^{\mathrm{A}}\left(1 - \frac{1}{2}\Gamma_{\mathrm{A}}\right) + \frac{1}{2}\Gamma_{\mathrm{A}} + \frac{1}{2}\Gamma_{\mathrm{AB}}^{\mathrm{A}}, \tag{5}$$

where the term $\Gamma^{\text{A}}_{\text{AB}}$ can be referred to as the partial cross-breed ancestral relationships of breed A. And $\mathbf{A}^{\text{A}^\Gamma}_{\text{AB,A}_{ij}}$ is the transpose of $\mathbf{A}^{\text{A}^\Gamma}_{\text{A,AB}_{ij}}$.

The off-diagonal elements of $\mathbf{A}^{\text{A}^\Gamma}_{\text{AB,AB}_{ij}}$ is similar to $\mathbf{A}^{\text{A}^\Gamma}_{\text{A,AB}_{ij}}$:

$$\begin{aligned} a^{\text{A}^\Gamma}_{\text{AB,AB}_{ij}} &= \frac{1}{2}\left(a^{\text{A}^\Gamma}_{\text{AB,AB}_{f(i)j}} + a^{\text{A}^\Gamma}_{\text{AB,AB}_{m(i)j}}\right) \\ &= \frac{1}{2}\left[a^{\text{A}}_{\text{AB,AB}_{f(i)j}}\left(1-\frac{1}{2}\Gamma_{\text{A}}\right)+\frac{1}{2}\Gamma_{\text{A}}+\frac{1}{2}\Gamma^{\text{A}}_{\text{AB}}\right. \\ &\qquad \left.+a^{\text{A}}_{\text{AB,AB}_{m(i)j}}\left(1-\frac{1}{2}\Gamma^{\text{A}}_{\text{B}}\right)+\frac{1}{2}\Gamma^{\text{A}}_{\text{B}}+\frac{1}{2}\Gamma^{\text{A}}_{\text{AB}}\right], \end{aligned} \tag{6}$$

where the term $\Gamma^{\text{A}}_{\text{B}}$ represents the special partial within-breed ancestral relationships. It is a part of the within-breed ancestral relationships of breed B, measured only by the information from breed A. It is obviously that $\Gamma^{\text{A}}_{\text{B}}$ is equal to 0. Therefore, this formula can be written as:

$$a^{\text{A}^\Gamma}_{\text{AB,AB}_{ij}} = a^{\text{A}}_{\text{AB,AB}_{ij}}\left(1-\frac{1}{2}\Gamma_{\text{A}}\right)+\frac{1}{4}\Gamma_{\text{A}}+\frac{1}{2}\Gamma^{\text{A}}_{\text{AB}}. \tag{7}$$

The diagonal elements of $\mathbf{A}^{\text{A}^\Gamma}_{\text{AB,AB}_{ii}}$ is equal to $f^{\text{A}}_i + \frac{1}{2}a^{\text{A}^\Gamma}_{f(i)m(i)}$. Due to $a^{\text{A}^\Gamma}_{f(i)m(i)} = \Gamma^{\text{A}}_{\text{AB}}$ and $f^{\text{A}}_i = \frac{1}{2}$ in two-way crossbreeding systems, $a^{\text{A}^\Gamma}_{\text{AB,AB}_{ii}}$ can be written as:

$$a^{\text{A}^\Gamma}_{\text{AB,AB}_{ii}} = a^{\text{A}}_{\text{AB,AB}_{ii}}\left(1-\frac{1}{2}\Gamma_{\text{A}}\right)+\frac{1}{4}\Gamma_{\text{A}}+\frac{1}{2}\Gamma^{\text{A}}_{\text{AB}}. \tag{8}$$

In summary, $\mathbf{A}^{\text{A}^\Gamma}$ can be written as:

$$\begin{aligned} a^{\text{A}^\Gamma}_{\text{A,A}_{ij}} &= a^{\text{A}}_{\text{A,A}_{ij}}\left(1-\frac{1}{2}\Gamma_{\text{A}}\right)+\Gamma_{\text{A}}, \\ a^{\text{A}^\Gamma}_{\text{A,AB}_{ij}} &= a^{\text{A}}_{\text{A,AB}_{ij}}\left(1-\frac{1}{2}\Gamma_{\text{A}}\right)+\frac{1}{2}\Gamma_{\text{A}}+\frac{1}{2}\Gamma^{\text{A}}_{\text{AB}}, \\ a^{\text{A}^\Gamma}_{\text{AB,AB}_{ij}} &= a^{\text{A}}_{\text{AB,AB}_{ij}}\left(1-\frac{1}{2}\Gamma_{\text{A}}\right)+\frac{1}{4}\Gamma_{\text{A}}+\frac{1}{2}\Gamma^{\text{A}}_{\text{AB}}. \end{aligned} \tag{9}$$

Appendix B. Population and genomic architecture in simulation study

In this appendix, we present the population details and genomic architecture simulated in our study.

We utilized simulation data based on a two-way crossbreeding system (Figure 1) to validate the effectiveness of our proposed method. The first generation in the historical population comprised 100 females and 600 males, with the population size remaining constant for 500 generations. Then, the size decreased to 300 animals to generate linkage disequilibrium. In the last historical generation (G0), the population size

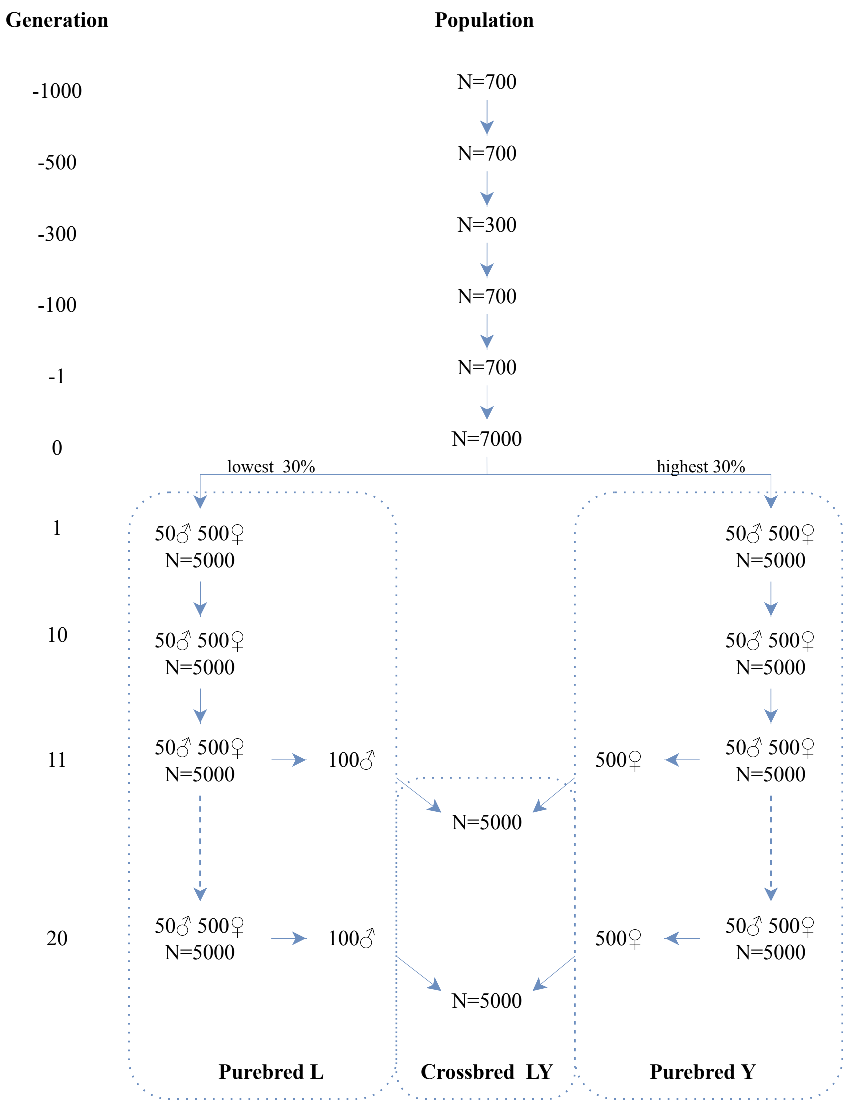

Figure 1. The simulation of breed L, breed Y and their crossbred descendants LY. The breed Y indicate positive selection based on simulated performance records and breed L indicate negative selection based on simulated performance. The crossbred LY is the crossbred descendants of animals from the last ten generation of the breed L and Y.

was increased to 7,000 animals for sampling. The proportion of males to females was maintained constant throughout all generations.

Two purebred populations (B and A) were established by sampling 50 males and 500 females in G0. The sample is based on phenotypes and has no overlap. The first generation of breed A was randomly sampled from the lowest 30% of phenotypes in G0, while breed B was sampled from the highest 30%. Within the purebred population, random mating continued for 20 generations (G1-G20). In each generation, 50 males and 500 females were randomly selected, and every female produced 10 offspring.

During generations 11 to 20 (G11-G20), a crossbred population (AB) was created by randomly selecting 100 males from breed A and 500 females from breed B. Individuals mated randomly across breeds. Each purebred female produced ten crossbred offspring. A total of ten crossbred generations (CG1-CG10) were simulated.

The genome comprised 18 chromosomes with 1.2 Morgan, each containing 500 QTL and 10,000 SNP. QTL positions were randomly simulated, while SNP positions were uniformly simulated. Biallelic SNP was simulated with uniformly distributed allele frequencies. During historical generations, the mutation rate of QTL and SNP was 2.5e-5, with no mutations occurring after the historical generations.

Following quality control, 5,472 QTL and 49,111 SNPs were segregated in G0 with a minor allele frequency greater than 0.05. Subsequently, all effects and phenotypes simulated by QMSim were discarded, and a new simulation was conducted.

The phenotypic and additive variances were set at 10 and 1, respectively, resulting in a constant narrow-sense heritability of 0.1. A random selection of 1,000 QTL from the 5,472 segregated QTL was made, and the additive values of each QTL were sampled from a standard normal distribution. The additive values were then scaled based on QTL allele frequencies to adjust the additive variance to 1.

The size of non-additive effects of QTL was assumed to depend on the size of additive effects at these QTLs. The dominance value (d) was calculated as the product of the dominance coefficient (δ) and the absolute value of additive value ($|a|$). The dominance coefficient, or the degree of dominance (Falconer 1996), was sampled from a normal distribution with a mean of 0.2, in line with empirical observations (Bennewitz and Meuwissen 2010; Sun and Mumm 2016). The standard deviation of the distribution was

based on the proportions of dominance and QTL allele frequencies. The proportion of dominance, defined as the ratio of dominance variance to the genotypic variance, was simulated as 0, 10%, 30%, or 50%. The additive and dominance variances were calculated as follows:

$$\sigma_{\mathrm{A}}^2 = \sum (2pq)\, \sigma_a^2 + \sum \left(2pq(q-p)^2\right)\sigma_d^2,$$
$$\sigma_D^2 = \sum \left((2pq)^2\right)\sigma_d^2. \tag{10}$$

Lastly, environmental effects were sampled from a normal distribution with a mean of 0 and a variable standard deviation, ensuring a constant phenotypic variance of 10.

Appendix C. The formula for the complex crossbreeding systems

In this appendix, we present the formula of our model for complex crossbreeding systems, which mainly means three-way or four-way crossbreeding systems and is expected in the production of pigs and poultry. In addition, those formulae are also suitable for the case that at least one parent of the individual is crossbred and both parents share the standard breed, such as crossbred animals mate with each other in the same crossbred population.

Additive relationship matrix

In three-way or more complex crossbreeding systems, it is easily proven that the pedigree-based additive partial relationship matrix $\mathbf{A}^{\mathrm{A}^{\Gamma}}$ is similar to that in two-way crossbreeding systems:

$$\mathbf{A}^{\mathrm{A}^{\Gamma}} = \mathbf{A}^{\mathrm{A}}\left(1 - \frac{1}{2}\Gamma_{\mathrm{A}}\right) + \mathbf{K}^{\mathrm{A}}\,\Gamma_{\mathrm{A}} + \textstyle\sum_{\mathrm{P}\neq\mathrm{A}} \mathbf{Q}_{\mathrm{AP}}^{\mathrm{A}}\,\Gamma_{\mathrm{AP}}^{\mathrm{A}}, \tag{11}$$

where the subscript P represents every purebred population except purebred A. The terms Γ_{A} and $\Gamma_{\mathrm{AP}}^{\mathrm{A}}$ denote within- and across-breed ancestral relationships, respectively. The recursive formulas for the element in $\mathbf{A}^{\mathrm{A}}$ are:

$$a_{ii}^{\mathrm{A}} = f_i^{\mathrm{A}} + \frac{1}{2}a_{f(i)m(i)}^{\mathrm{A}},$$
$$a_{ij}^{\mathrm{A}} = \frac{1}{2}\left(a_{f(i)j}^{\mathrm{A}} + a_{m(i)j}^{\mathrm{A}}\right), \tag{12}$$

where the element a_{ij}^{A} signifies the additive partial relationship between animals i and j; animal j is not a descendant of i. Additionally, the terms $f(i)$ and $m(i)$ represent the parents of animal i, and the term f_i^{A} denotes the breed A proportion of animal i.

Then, the coefficient matrices $\mathbf{K}^{\mathrm{A}}$ and $\mathbf{Q}_{\mathrm{LP}}^{\mathrm{A}}$ are different from that in two-way crossbreeding systems:

$$
\begin{aligned}
k_{ij}^{\mathrm{A}} &= f_{f(i)}^{\mathrm{A}} f_{m(i)}^{\mathrm{A}}, \\
q_{\mathrm{AP}_{ij}}^{\mathrm{P}} &= f_{f(i)}^{\mathrm{A}} f_{m(i)}^{\mathrm{P}} + f_{f(i)}^{\mathrm{P}} f_{m(i)}^{\mathrm{A}},
\end{aligned}
\tag{13}
$$

where the terms k_{ij}^{A} and $q_{\mathrm{AP}_{ij}}^{\mathrm{P}}$ are the element of the coefficient matrices.

Dominance relationship matrix

In three-way or more complex crossbreeding systems, especially in the case of the crossbred animals sharing the standard breed mating with each other, the off-diagonal elements of the pedigree-based dominance partial relationship matrix $\mathbf{D}^{\mathrm{A}}$ must be adjusted to:

$$
d_{ij}^{\mathrm{A}} = \frac{\left(a_{f(i)f(j)} a_{m(i)m(j)}\right)^{\mathrm{A}} + \left(a_{f(i)m(j)} a_{m(i)f(j)}\right)^{\mathrm{A}}}{4}, \tag{14}
$$

where the element $a_{f(i)f(j)}$ signifies the additive relationship between animals $f(i)$ and $f(j)$, and the animals $f(i)$ or $f(j)$ represent the parents of animal i or j.

Then, the right-hand term of d_{ij}^{A} is defined as:

$$
\left(a_{f(i)f(j)} a_{m(i)m(j)}\right)^{\mathrm{A}} = a_{f(i)f(j)}^{\mathrm{A}} a_{m(i)m(j)}^{\mathrm{A}} + a_{f(i)f(j)}^{\mathrm{A}} \sum_{\mathrm{P}} k^{\mathrm{P}} a_{m(i)m(j)}^{\mathrm{P}} + a_{m(i)m(j)}^{\mathrm{A}} \sum_{\mathrm{P}} k^{\mathrm{P}} a_{f(i)f(j)}^{\mathrm{P}}, \tag{15}
$$

where the term $k^{\mathrm{P}} = \frac{f_{ij}^{\mathrm{A}}}{f_{ij}^{\mathrm{A}} + f_{ij}^{\mathrm{P}}}$, and superscript P denotes all breeds excluding breed A.

References

Bennewitz J and Meuwissen T. 2010. The distribution of QTL additive and dominance effects in porcine F2 crosses. *Journal of Animal Breeding and Genetics*. **127**: 171–179.

Falconer DS. 1996. Introduction to quantitative genetics. In. Pearson Education India.

Legarra A, Christensen OF, Vitezica ZG, Aguilar I, and Misztal I. 2015. Ancestral relationships using metafounders: finite ancestral populations and across population relationships. *Genetics*. **200**: 455–468.

Sun X and Mumm RH. 2016. Method to represent the distribution of QTL additive and dominance effects associated with quantitative traits in computer simulation. *BMC bioinformatics*. **17**: 1–15.
